# Role of nonpneumoniae mycoplasma in the pathogenesis of ventilator-associated pneumonia: an *in vitro *assessment

**DOI:** 10.1186/cc14071

**Published:** 2014-12-03

**Authors:** TJ Nolan, AC Morris, A Rossi, T Walsh

**Affiliations:** 1 Centre for Inflammation Research, Queens Medical Research Centre, Little France, Edinburgh, UK

## Introduction

**Figure 1:**
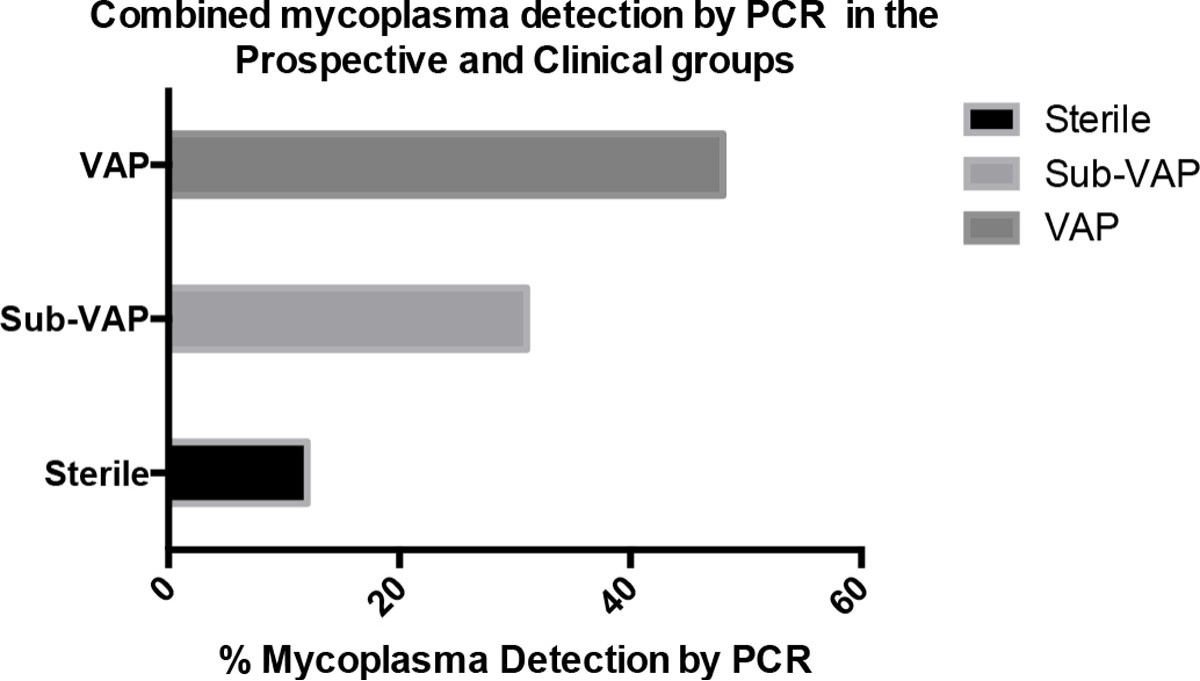
**Combined mycoplasma detection by PCR in the prospective and clinical groups**.

Mycoplasma organisms are the smallest bacteria capable of self-replication [1] and include species capable of causing disease (for example, *Mycoplasma pneumoniae, Mycoplasma genitalium*) as well as those that are generally thought to exist synergistically with their human host (for example, *Mycoplasma salivarium*). The Edinburgh critical care group (Prof TW/ACM) has recently identified a high prevalence of *M. salivarium *in the bronchoalveolar lavage washings from patients with confirmed and suspected ventilator-associated pneumonia (VAP) (Figure 1) [2]. The aim of this study was to examine the effect of *M. salivarium *on human immune cells *in vitro*. Specifically, we measured cytokine production and phagocytosis activity in response to *M. salivarium *exposure.

## Methods

Whole human blood was obtained from healthy donor volunteers and cell types were isolated using diffusion gradients and magnetic labeling as appropriate. Monocytes and macrophages were incubated with *M. salivarium *for 24 hours before a subsequent LPS stimulus. Macrophage phagocytosis assays were conducted after exposure times of 60 minutes and 24 hours to *M. salivarium*. Cytokines were measured using ELISA and human cytokine bead array kits.

## Results

**Figure 2:**
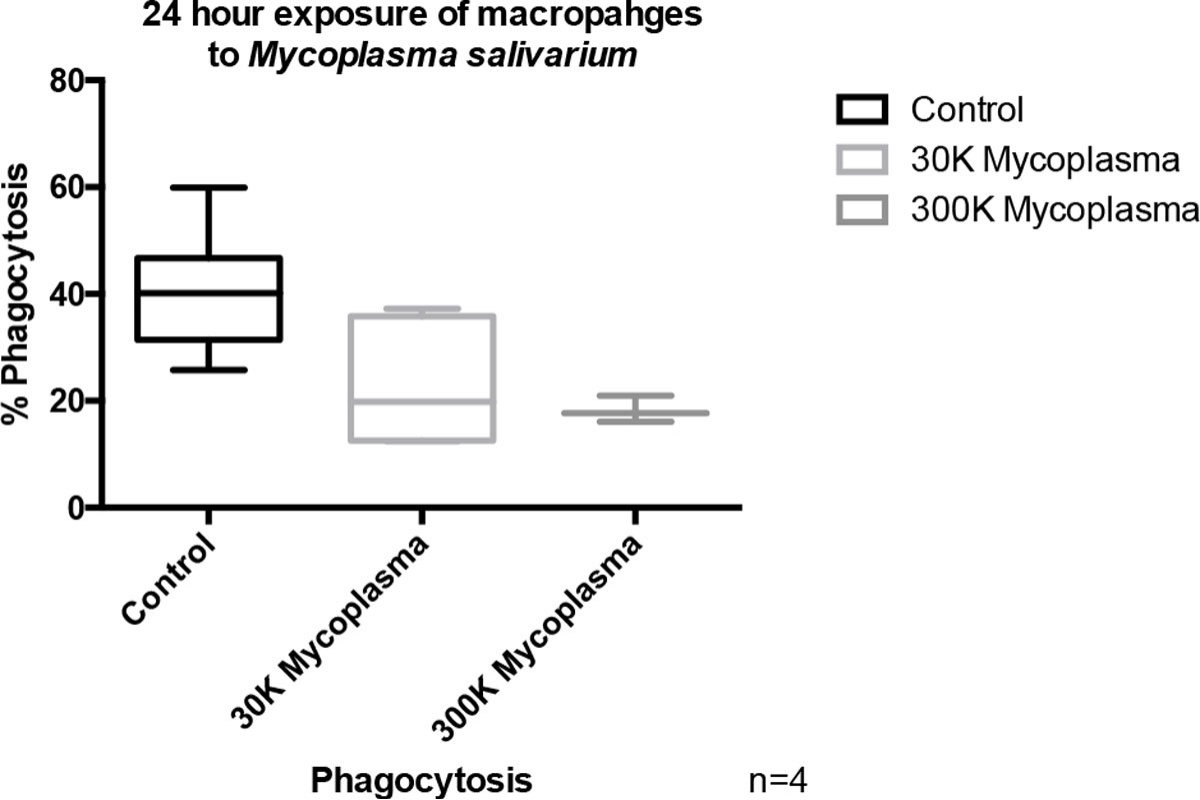
Twenty-four-hour exposure of macrophages to *Mycoplasma salivarium*.

**Figure 3:**
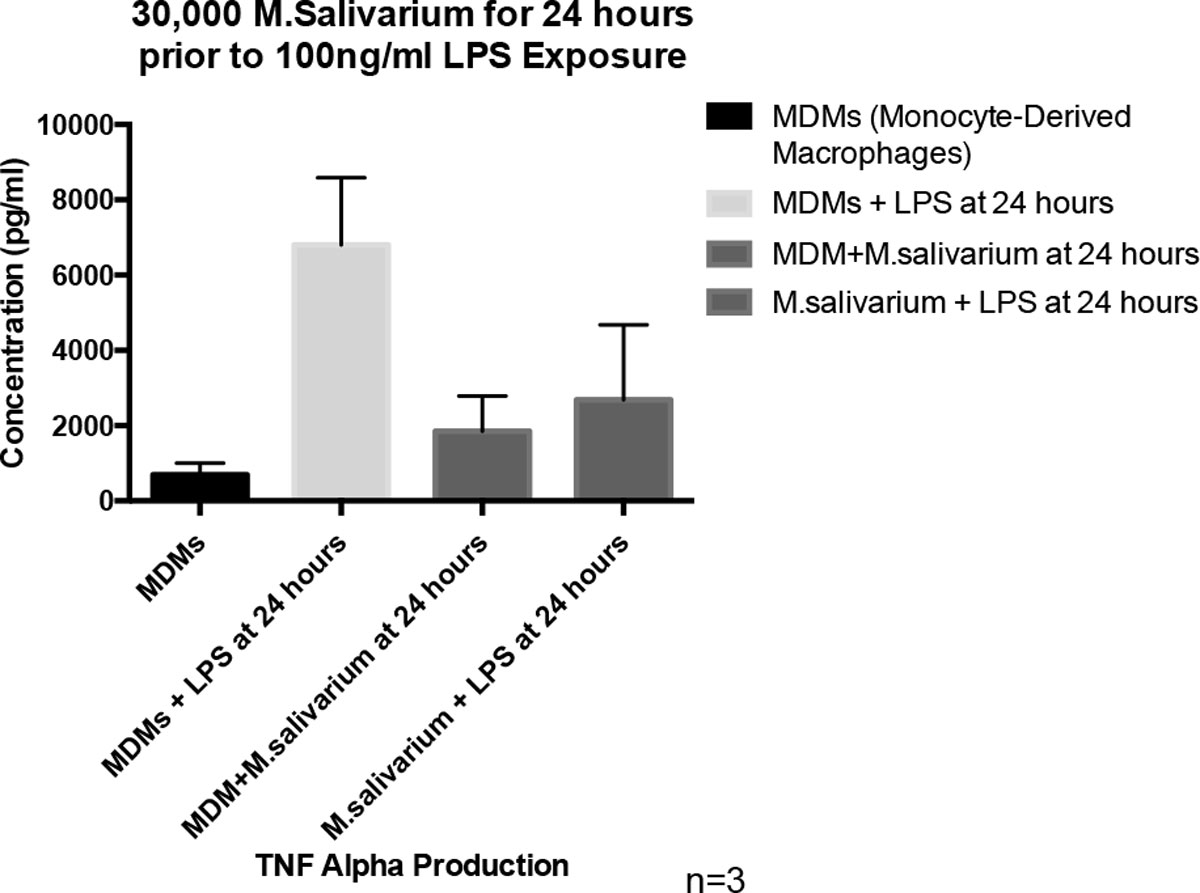
***Mycoplasma salivarium *(30,000) for 24 hours prior to 100 ng/ml LPS exposure**.

**Figure 4:**
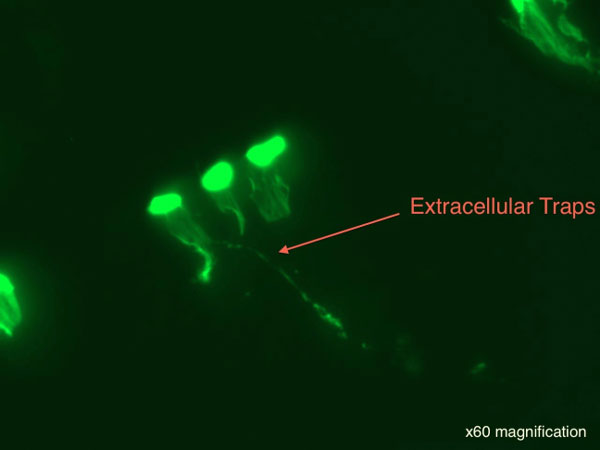


There was a statistically significant decrease in phagocytosis between control cells and the macrophages exposed to both a low titer of *M. salivarium *(*P *value 0.018) and a medium titer of *M. salivarium *(*P *value 0.011) after 24 hours of exposure (Figure 2). There was a statistically significant decrease in phagocytosis activity between the macrophages exposed to the medium titer of *M. salivarium *for 24 hours versus 60 minutes (*P *value 0.013). Exposure of macrophages to mycoplasma resulted in decreased release of TNFα after a subsequent LPS stimulus (Figure 3). To our knowledge, this is the first time extracellular traps have been induced in macrophages in response to *M. salivarium *(Figure 4).

## Conclusion

Although further research is needed, it is interesting that the presence of *M. salivarium *caused an anti-inflammatory effect as well as impaired antigen presentation secondary to impaired phagocytosis. This could be consistent with the better outcome in mechanically ventilated patients that did not have *M. salivarium *bacteria detected in their bronchoalveolar lavage washings. Extracellular traps contribute to microbial containment by forming a physical barrier composed of chromatin and cytoplasmic proteins to enhance antimicrobial synergy while minimizing damage to host tissues [3]. It is interesting that *M. salivarium *induced extracellular traps.
